# Bioactive adrenomedullin as a point-of-care biomarker in emergency department patients with suspected severe infections: an exploratory analysis

**DOI:** 10.1186/s12879-026-12951-1

**Published:** 2026-02-28

**Authors:** Constantin Maier-Stocker, Julian Hupf, Jillena Zinsser-Krys, Markus Zimmermann, Frank Hanses

**Affiliations:** 1 Department of Emergency Medicine, University Hospital Regensburg, Regensburg, Germany; 2 Department for Infection Control and Infectious Diseases, University Hospital Regensburg, Regensburg, Germany

**Keywords:** Adrenomedullin, Infection, Sepsis, Emergency department, Point-of-care

## Abstract

**Background:**

Early identification and treatment of patients with severe infections or sepsis remains challenging, and suitable biomarkers are lacking. This prospective exploratory pilot study aimed to evaluate the potential of point-of-care bioactive adrenomedullin (POC bio-ADM) in predicting bacteremia in patients presenting to the emergency department (ED) with suspected severe infections. Secondary objectives included its association with any infection, in-hospital mortality, intensive care unit (ICU) admission, and ICU length of stay.

**Methods:**

In this prospective exploratory pilot cohort study, adult patients presenting to the ED of a German university hospital between December 2022 and September 2023 were enrolled. POC bio-ADM levels were analyzed using univariate and multivariable binary logistic regression and compared to established sepsis biomarkers (PCT, CRP, lactate).

**Results:**

47 patients were included in the final analysis. POC bio-ADM was positive (> 45 pg/ml) in 38.3% (95% CI: 25.8–52.6%). Bacteremia was found in 34.0% (95% CI: 22.2–48.3%), and 78.7% (95% CI: 65.1–88.8%) had an infection. Positive POC bio-ADM was associated with significantly higher rates of bacteremia (55.6% vs. 20.7%). In the multivariable analysis, a positive POC bio-ADM was associated with an odds ratio (OR) of 5.318 (95% CI: 1.217–23.243) for bacteremia. Attributable in-hospital mortality was higher in patients with elevated POC bio-ADM (44.4% vs. 3.6%; OR: 21.6, 95% CI: 2.4-195.3).

**Conclusion:**

This small, single-centre pilot exploratory study suggests that POC bio-ADM may help identify ED patients with suspected severe infection at increased risk of bacteremia and in-hospital mortality; these findings are hypothesis-generating and require confirmation in larger cohorts.

**Supplementary Information:**

The online version contains supplementary material available at 10.1186/s12879-026-12951-1.

## Background

Suspected infections are a common cause of ED visits. The scenarios range from minor or more severe infections to sepsis and septic shock. Sepsis is a life-threatening organ dysfunction caused by a dysregulated host response to infection affecting approximately 49 million people globally with 11 million sepsis-related deaths annually [1]. Recent data suggest sepsis incidence continues to rise globally. Diagnosis and treatment of sepsis is time critical and delayed recognition is associated with significant additional morbidity and mortality [2]. It has been suggested that each hour of delay in appropriate antibiotic administration increases mortality by as much as 7.6% in patients with septic shock [3]. Many sepsis patients, especially those with community acquired infections, initially present to the emergency room. Early diagnosis and management of sepsis, however, remains a challenge for emergency care providers [4]. The economic burden of sepsis is substantial, with estimated costs exceeding $62 billion annually in the United States alone [5]. Clinical scores such as the quick Sepsis-related organ failure assessment (qSOFA) leave room for improvement and timely available biomarkers that are accepted and validated are lacking [6]. A recent study of 1477 patients in emergency departments demonstrated that biomarkers, including bio-ADM, can significantly improve sepsis diagnostics when added to clinical scores such as qSOFA [7]. In this context, bioactive adrenomedullin (bio-ADM) has been studied as a promising biomarker candidate for sepsis and severe infections.

Adrenomedullin (ADM) is a peptide hormone that circulates freely and consists of 52 amino acids. It is known to be involved in several pathophysiological conditions, including sepsis. ADM is produced from a larger precursor peptide, Pro-ADM and is synthesized and released by most mammalian tissues under conditions of hypoxia, inflammation and oxidative stress [8]. Upon release, ADM exhibits vasodilatory properties and plays a crucial role in modulating the function of the endothelial barrier, making it both a plausible biomarker as well as a potential therapeutic target [9]. Previous research has detected increased concentrations of ADM in the plasma of patients suffering from sepsis. Moreover, a significant correlation has been noted between the levels of ADM and both the severity of sepsis and its associated mortality rate [10]. Bio-ADM was found to be an independent factor for mortality and organ failure in ICU patients with sepsis after adjusting for morbidity [11]. Bio-ADM levels were also significantly higher in patients with septic shock and non-survivors with test characteristics similar to the Sepsis-related organ failure assessment (SOFA) score for predicting sepsis mortality [12]. In the ED, patients with sepsis and elevated ADM levels had higher odds for mortality, multi organ failure, ICU admission and reduced odds for discharge from the ED. Bio-ADM was better in predicting 28d-mortality than either CRP, lactate or creatinine [13]. At the same time, point-of-care testing technologies may enable swift and almost real-time biomarker testing in patients evaluated for sepsis in the ED. Large multicentre cohorts such as the prospective AdrenOSS-1 study by Mebazaa et al. [14] and ICU-based work by Caironi et al. [15] have shown that circulating bio-ADM is strongly associated with vasopressor use, organ failure and short-term mortality in sepsis and septic shock, but have not addressed its point-of-care use in unselected ED patients with suspected severe infection.

Beyond bio-ADM, mid-regional pro-adrenomedullin (MR-proADM) has been evaluated as a prognostic biomarker in sepsis for more than a decade. A recent systematic review and meta-analysis found that MR-proADM shows moderate to good prognostic accuracy for mortality and organ failure in sepsis and septic shock, while emphasising heterogeneity between studies and uncertainty about optimal cut-offs [16].

The hypothesis of this study was that POC bio-ADM can predict bacteremia in patients presenting to the ED with suspected severe infections.

## Methods

This pilot study was designed as a prospective cohort study without predefined sample size comprising a convenience sample. The study was conducted at the ED of the University Hospital Regensburg, Germany. Recruitment continued until no further POC bio-ADM test kits were available, as the manufacturer had discontinued production and could not provide additional test kits for study use. Informed consent was obtained from all participants or their legal representatives prior to inclusion in the study. The study was approved by the local ethic committee (Vote # 23-3397-101). Study results are presented according to the STARD guidelines [17].

Patients aged 18 years and older who presented to the ED between December 2022 and September 2023 were screened for inclusion if the ED clinician suspected a severe infection after the initial examination. Inclusion criteria required either an elevated lactate level ≥ 30 mg/dl in the initial blood gas analysis, a National Early Warning Score 2 (NEWS-2) ≥ 4 or red flags within the NEWS-2 upon admission to the ED. Red flags were defined as respiratory rate ≤ 8/min or ≥ 25/min, oxygen saturation on room air ≤ 91%, systolic blood pressure ≤ 90 mmHg or ≥ 220 mmHg, heart rate ≤ 40/min or ≥ 131/min, body temperature ≤ 35 °C, or impaired consciousness. Blood samples were collected as part of routine diagnostic assessments in the ED. To be included in the final analysis, patients were required to have at least two sets of blood cultures drawn in the ED. Patients were excluded from the study if they exhibited overt signs of shock. These inclusion criteria were chosen to capture patients with intermediate to high risk of severe infections while excluding both low-risk patients and those with clinical signs of septic shock.

Bio-ADM levels were assessed in whole blood (EDTA blood) using point-of-care testing with a NexusDx IB10 provided by SphingoTec GmbH, Hennigsdorf, Germany. Quality control procedures included regular calibration checks and adherence to the manufacturer’s specifications. The test setup allowed the result of the bio-ADM-testing to be read after 20 min. In the initial test batch, the lower limit of detection (LOD) for bio-ADM was 45 pg/ml, whereas in the subsequent batches, it was 30 pg/ml. For the primary analysis, a cut-off > 45 pg/ml was chosen to align with the highest lower LOD of the assay. In sensitivity analyses, we additionally evaluated a cut-off defined by the batch-specific LOD (> 30 pg/ml or > 45 pg/ml) and explored a Youden-index-derived threshold from the ROC curve for bacteremia (46.1 pg/ml); as no measurements fell between 45 pg/ml and 46.1 pg/ml, the Youden-derived threshold yielded an identical classification of patients compared with the primary 45 pg/ml cut-off.

All other biomarkers were analyzed as part of routine procedures in the hospital’s certified laboratory.

The primary endpoint of the study was the rate of bacteremia, defined as at least one set of positive blood cultures, because this constitutes an objective and clinically meaningful outcome in ED patients with suspected severe infection. However, many patients with severe infection or sepsis remain culture-negative; therefore, secondary endpoints comprised the diagnosis of any infection via positive microbiologic, cytologic, or pathologic findings in sterile materials. Additionally, morphological evidence of infection on imaging studies was considered a secondary endpoint. The diagnosis of any infection was adjudicated by two physicians based on microbiological, imaging and clinical data, with one reviewer blinded to POC bio-ADM results. In case of uncertainty, the final discharge letter was used to determine whether an infection had been diagnosed and treated. Ambiguous cases were resolved by discussion between two physicians and, if needed, consultation of an Infectious disease specialist. Retrospective analysis was performed on the results of routine diagnostics conducted in the ED. Furthermore, patients’ medical histories, comorbidities, and vital signs upon ED admission were documented. NEWS-2, Emergency Severity Index (ESI), Charlson Comorbidity Index, Pitt Bacteremia Score, qSOFA, and SOFA were calculated to characterize the study cohort and explore potential correlations with POC bio-ADM levels.

Sepsis was defined according to the Sepsis-3 definition as a clinically documented diagnosis of infection and a change of two or more points in the baseline SOFA score. For the calculation of SOFA scores, patients with no known pre-existing organ dysfunction were assumed to have a baseline score of zero, and oxygen saturation (SpO2) was used instead of partial pressure of oxygen (PaO2).

Patients were followed-up until discharge with regard to their length of hospital stay, in-hospital mortality, ICU admission rates, and ICU length of stay using hospital records. Attributable in-hospital mortality, defined as the proportion of in-hospital deaths attributable to the infection that led to the patient’s presentation to the emergency department, was independently adjudicated by two physicians based on the final hospital discharge diagnoses, with one reviewer blinded to POC bio-ADM concentrations. In the single case of disagreement, a third physician made the final classification.

Blood culture contaminants were defined a priori as typical skin flora without compatible clinical or imaging findings and were classified as non-bacteremic. Severe infections and sepsis without documented bacteremia were not captured by the primary endpoint but were reflected in the secondary endpoint, the diagnosis of any infection, and in the Sepsis-3-based description of the cohort’s illness severity.

Statistical analyses were conducted using IBM SPSS Statistics 29.0. Statistical significance was defined as *p* < 0.05. Effect sizes are reported with 95% confidence intervals (CI). The Pearson’s chi-square test was used to compare categorical data between groups, while the Wilcoxon rank-sum test (Mann-Whitney U test) was employed for comparing continuous variables between two groups. Multivariable binary logistic regression analysis was applied to assess the primary outcome, with covariates included if they exhibited a different distribution (*p* < 0.1) concerning the primary outcome. Given the small number of events (16 bacteremias), multivariable logistic regression was restricted to a maximum of two covariates (age and Charlson Comorbidity Index) in addition to POC bio-ADM to limit overfitting. All multivariable analyses are exploratory. Results from the regression analyses are reported as odds ratios (OR) with corresponding 95% confidence intervals. To address model stability given the low events-per-variable ratio (EPV ≈ 5; 16 events / 3 parameters), 1,000 bootstrap resamples (simple random sampling with replacement) were performed on the primary multivariable model.

To evaluate the diagnostic efficacy of POC bio-ADM and to compare it with other biomarkers, sensitivity, specificity, positive predictive value, and negative predictive value were calculated for each test.

Receiver operating characteristic (ROC) curves and corresponding areas under the curve (AUCs) were calculated for POC bio-ADM, PCT, CRP and lactate with respect to the presence of bacteremia and the diagnosis of infection of any type. Diagnostic performance was summarised at pre-specified cut-offs (POC bio-ADM 45 pg/ml, PCT 0.5/1.0 ng/ml, CRP 10 mg/l, lactate 20 mg/dl) and, in sensitivity analyses, at POC bio-ADM batch-specific LOD cut-off (> 30 pg/ml and > 45 pg/ml) and Youden-index optimized cut-offs for each biomarker and both outcomes (Supplement S.1 and S.2).

In cases where a variable had missing values for a patient, the individual was excluded from the respective variable analyses.

Sample size calculations were performed with G*Power (version 3.1.9.7, University of Düsseldorf, Germany) using an a priori z-test for logistic regression with a dichotomous predictor and dichotomous outcome.

## Results

### Participants

**Fig. 1:**
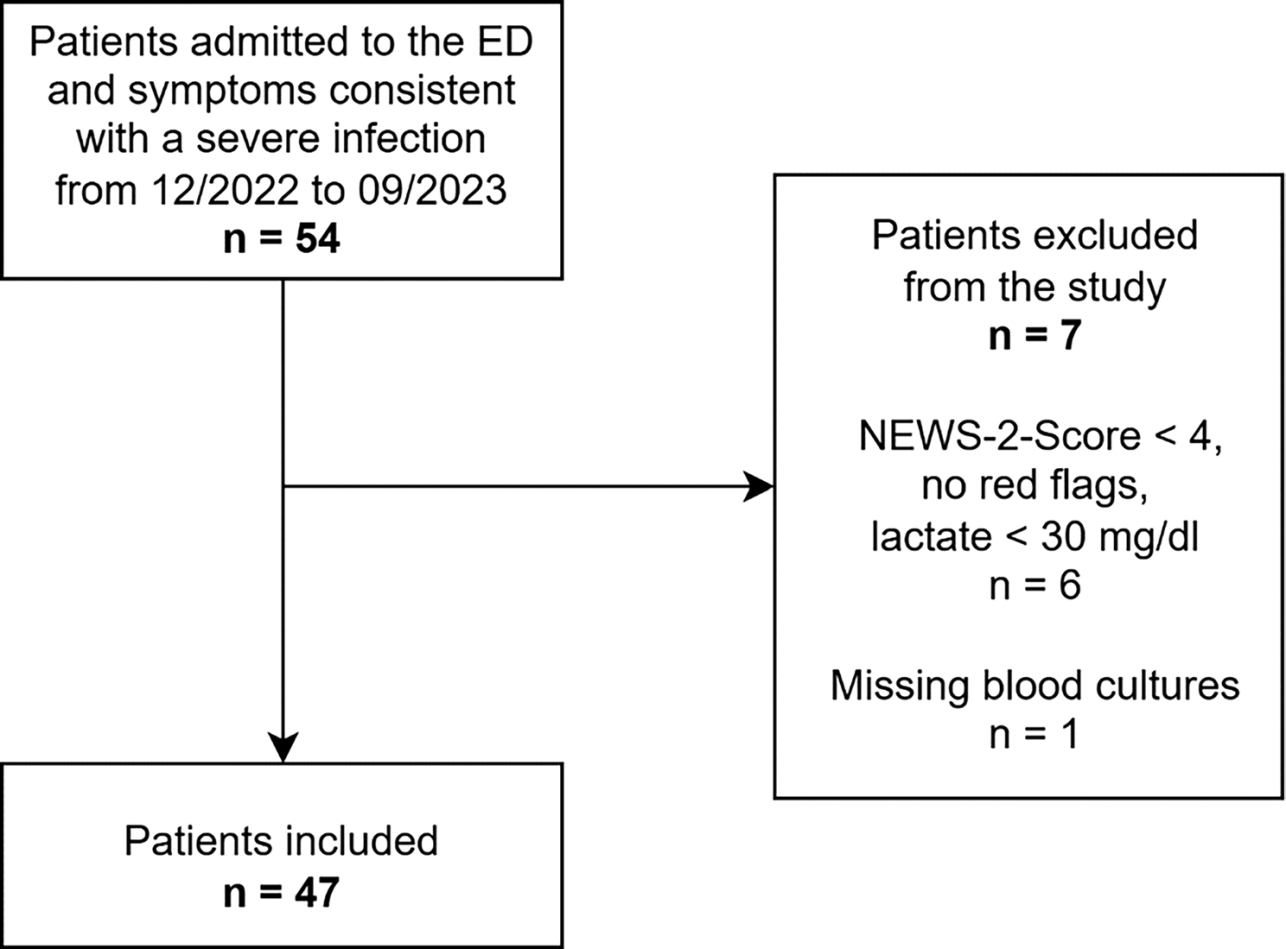
Flow chart of the study population selection including patient recruitment and exclusion criteria

A total of 54 patients were recruited into the study. Seven of these patients were excluded from the analysis: six patients did not meet the inclusion criteria (lactate < 30 mg/dl. NEWS-2 < 4 and no red flags within NEWS-2), and one patient was excluded because no blood cultures were obtained in the ED. Consequently, the final analysis included 47 patients, as illustrated in Fig. 1.

Patient characteristics of the study population are presented in Table 1. Among the 47 patients included, 35 (74.5%) were male. The median age of the cohort was 68 years. The cohort was highly acute, with a median NEWS-2 of 7 (range 1–17, IQR 5–8) and 76.6% (36/47) of the patients meeting Sepsis-3 criteria (SOFA score of two or more points) and 23.4% (11/47) with a qSOFA score > 1, consistent with the observed high in-hospital mortality. Table 1 also summarizes the mean values of biomarkers and outcome parameters investigated. The POC bio-ADM levels ranged from 45 to 500 pg/ml (mean 102.3 pg/ml). 38.3% (18/47, 95% CI: 25.8–52.6%) of the patients tested positive for POC bio-ADM (cut-off > 45 pg/ml, lower limit of detection).

Positive blood cultures were observed in 34.0% (16/47, 95% CI: 22.2–48.3%) of the patients, while 78.7% (37/47, 95% CI: 65.1–88.8%) of the patients included had an infection of any type. The following pathogens were detected in the blood cultures obtained: *Acinetobacter sp.* (1x), *Citrobacter sp.* (1x), *Clostridium perfringens* (1x), *Clostridium ramosum* (1x), *Escherichia coli* (4x), *Pseudomonas aeruginosa* (1x), *Serratia marcescens/ureilytica* (1x), *Staphylococcus aureus* (1x), Coagulase-Negative Staphylococci (3x), *Streptococcus pneumoniae* (3x), *Streptococcus pyogenes* (1x), *Yersinia enterocolitica* (1x).

Overall in-hospital mortality among the study cohort was 26.1%, the attributable in-hospital mortality rate was 19.6%. One patient was lost to follow-up due to transfer to another hospital, resulting in a lack of available information.

**Table 1:** Baseline characteristics

Male (*n*)	35	(74.5%)
Age (median)	68 years	(18–96, IQR 50–76)
ESI 2 (n)	41	(87.2%)
ESI 3 (n)	6	(12.8%)
Charlson (*n* = 46) (median)	3	(0–16, IQR 0–5)
NEWS-2 (median)	7	(1–17, IQR 5–8)
qSOFA (median)	1	(0–2, IQR 1–1)
SOFA (median)	3	(0–11, IQR 2–5)
Pitt (median)	1	(0–6, IQR 0–2)
POC bio-ADM (mean)	102.3 pg/ml	(45–500, SD 121.6)
PCT (*n* = 46) (mean)	11.53 ng/ml	(0.06–100.00, SD 25.84)
CRP (mean)	203.1 mg/l	(3.7–682.0, SD 128.3)
Lactate (*n* = 44) (mean)	25 mg/dl	(6-124, SD 21)
Blood cultures positive (n)	16	(34.0%)
Any infection (n)	37	(78.7%)
Overall in-hospital mortality (*n* = 46) (n)	12	(26.1%)
Attributable in-hospital mortality (*n* = 46) (n)	9	(19.6%)
Length of stay (*n* = 46) (median)	12 days	(0–61, IQR 7–25)
ICU admission (*n* = 46) (n)	22	(46.8%)
ICU length of stay (*n* = 21) (median)	6 days	(1–48, IQR 2–27)

### Biomarker

A positive POC bio-ADM (cut-off > 45 pg/ml) was found to be significantly correlated with an increased likelihood of positive blood cultures. Conversely, none of the other investigated biomarkers demonstrated a statistically significant association with an increased incidence of positive blood cultures (Table 2). Compared to other biomarkers, a higher incidence of any infection was observed in the ED when POC bio-ADM tested positive. In all patients with an elevated POC bio-ADM level, an infection of any type was diagnosed. Additionally, elevated lactate levels (> 20 mg/dl) were significantly associated with the diagnosis of an infection (Table 2).

**Table 2:** Rates of positive blood cultures and rates of infections of any type in relation to positive or negative test results for different biomarkers; RR (relative risk); 95% CI in round brackets; *p*-values from Pearson’s chi-square test

	Biomarker negative	Biomarker positive	RR	*p*-value
**Blood cultures positive**				
**POC bio-ADM** [*n* = 47] **(cut-off > 45 pg/ml)**	**20.7% ** (9.8–38.4%)	**55.6% ** (33.7–75.4%)	**2.69** (1.18–6.12)	**0.014**
PCT [*n* = 46] (cut-off > 0.5 ng/ml)	22.2% (6.3–54.7%)	37.8% (24.1–53.9%)	1.70 (0.47–6.17)	0.378
PCT [*n* = 46] (cut-off > 1.0 ng/ml)	20.0% (7.0-45.2%)	41.9% (26.4–59.2%)	2.10 (0.70–6.26)	0.143
CRP [*n* = 47] (cut-off > 10 mg/l)	66.7% (20.8–93.9%)	31.8% (20.0-46.6%)	0.48 (0.19–1.19)	0.218
Lactate [*n* = 44] (cut-off > 20 mg/dl)	25.0% (12.7–43.4%)	50.0% (28.0–72.0%)	2.00 (0.89–4.49)	0.092
**Any Infection**				
**POC bio-ADM** [*n* = 47] **(cut-off > 45 pg/ml)**	**65.5% ** (47.3–80.1%)	**100% ** (82.4–100%)	**1.53** (1.17–1.99)	**0.005**
PCT [*n* = 46] (cut-off > 0.5 ng/ml)	55.6% (26.7–81.1%)	83.8% (68.9–92.3%)	1.51 (0.83–2.75)	0.066
PCT [*n* = 46] (cut-off > 1.0 ng/ml)	60.0% (35.7–80.2%)	87.1% (71.1–94.9%)	1.45 (0.94–2.24)	0.037
CRP [*n* = 47] (cut-off > 10 mg/l)	100% (43.9–100%)	77.3% (63.0-87.2%)	0.77 (0.66–0.91)	0.352
Lactate [*n* = 44] (cut-off > 20 mg/dl)	67.9% (49.3–82.1%)	93.8% (71.7–98.9%)	1.38 (1.04–1.84)	0.049

Patients who tested positive for POC bio-ADM were significantly older and had a higher SOFA score than those with negative results. Although patients with more comorbidities tended to have a higher likelihood of positive POC bio-ADM, the difference did not reach statistical significance. Furthermore, the body temperature and other calculated risk scores were not correlated with the level of POC bio-ADM (Table 3).

**Table 3:** Covariates; *p*-values from Mann-Whitney U test for continuous variables and chi-square test for categorical variables

POC bio-ADM	negative*	positive*	*p*-value
Age (in years, median) [*n* = 47]	56	75	**0.002**
Sex (male) [*n* = 47]	72.4%	77.8%	0.682
NEWS-2 (median) [*n* = 47]	7	6	0.316
Charlson (median) [*n* = 46]	2	4	0.053
Pitt (median) [*n* = 47]	0	2	0.159
qSOFA (median) [*n* = 47]	1	1	0.479
SOFA (median) [*n* = 47]	2	5	**< 0.001**
ESI (median) [*n* = 47]	2	2	0.130
Temperature (°C, mean) [*n* = 46]	38.1	37.8	0.119

*POC bio-ADM cut-off > 45 pg/ml

The univariate association between positive POC bio-ADM and the rate of positive blood cultures remained statistically significant in the multivariable binary logistic regression analysis (OR: 5.318, 95% CI: 1.217–23.243). The variables age and Charlson Comorbidity Index were not found to be associated with the rate of positive blood cultures in this model (Table 4).

**Table 4:** Multivariable binary logistic regression analysis in reference to positive blood cultures

Blood cultures positive	OR	95% CI	*p*-value
**POC bio-ADM> 45 pg/ml**	**5.318**	**1.217–23.243**	**0.026**
Age	1.001	0.966–1.001	0.068
Charlson	1.182	0.808–1.182	0.814

Bootstrap validation (*n* = 1,000 resamples) confirmed internal stability of the association between positive POC bio-ADM and bacteremia: the bootstrap estimate for POC bio-ADM was B = 1.671 (OR 5.318), with a bias of + 0.182, and a 95% CI (B) 0.207–4.073 (95% CI (OR) 1.23–59.1), *p* = 0.033 (Table 5).

**Table 5:** Bootstrap validation (*n* = 1,000 resamples) of multivariable logistic regression for bacteremia

Variable	B	OR (Exp(B))	Bootstrap Bias	95% CI (OR)	*p*-value
**POC bio-ADM> 45 pg/ml**	**1.671**	**5.318**	**+ 0.182**	**1.23–59.1**	**0.033**
Age	-0.017	0.984	-0.001	0.96-1.00	0.065
Charlson	-0.023	0.977	-0.023	0.68–1.19	0.796

The median hospital length of stay, the ICU admission rate, and the ICU length of stay did not show significant differences between the POC bio-ADM groups (with a POC bio-ADM cut-off greater than 45 pg/ml) in this cohort. However, both the overall and the attributable in-hospital mortality of patients with a positive POC bio-ADM in the ED were significantly higher compared to patients with a negative POC bio-ADM (Table 6). The odds of attributable in-hospital mortality were substantially increased in patients with elevated PoC bio-ADM (OR 21.6, 95% CI: 2.389-195.298; 44.4% vs. 3.6%).

**Table 6:** Comparison of bio-ADM negative and positive patients: mortality and admission data, p-values from Mann-Whitney U test for continuous variables and chi-square test for categorical variables

POC bio-ADM	negative*	positive*	*p*-value
Median in-hospital length of stay (days) [*n* = 46]	11	16	0.180
Overall in-hospital mortality [*n* = 46]	10.7%	50.0%	**0.003**
Attributable in-hospital mortality [*n* = 46]	3.6%	44.4%	**< 0.001**
ICU-admissions [*n* = 22/46]	42.9%	55.6%	0.400
Median ICU-length of stay (days) [*n* = 21/46]	7	4	0.651

*POC bio-ADM cut-off> 45 pg/ml (1 Patient lost to follow-up)

### Predictive value and clinical utility

POC bio-ADM exhibited the highest specificity and positive predictive values among the tested biomarkers, albeit with only moderate sensitivity. It demonstrated a specificity of 74.2%, a positive predictive value (PPV) of 55.6%, a negative predictive value (NPV) of 79.3% and a sensitivity of 62.5%. Regarding the diagnosis of any type of infection, POC bio-ADM maintained a specificity and PPV of 100%, but its sensitivity remained low at 48.6%. For bacteremia, the area under the curve (AUC) was 0.602 for POC bio-ADM, 0.707 for PCT, 0.559 for CRP, and 0.668 for lactate. For any infection, AUCs were numerically higher for POC bio-ADM and PCT than for CRP and lactate, although confidence intervals were wide and overlapped, reflecting the small sample (summarized in Table 7).

**Table 7:** Biomarkers performance metrics; 95% CI in round brackets; ROC Curves provided in the supplement S.3 and S.4

	Sensitivity	Specificity	PPV	NPV	AUC
**Bacteremia**					
POC bio-ADM > 45 pg/ml	0.625	0.742	0.556	0.793	0.602 (0.424–0.779)
PCT > 0.5 ng/ml	0.875	0.233	0.378	0.778	0.707 (0.544–0.871)
PCT > 1.0 ng/ml	0.813	0.400	0.419	0.800	
CRP > 10 mg/l	0.875	0.032	0.318	0.333	0.559 (0.374–0.745
Lactate > 20 mg/dl	0.533	0.724	0.500	0.750	0.668 (0.490–0.846)
**Any Infection**					
POC bio- ADM > 45 pg/ml	0.486	1.000	1.000	0.345	0.816 (0.690–0.942)
PCT > 0.5 ng/ml	0.861	0.400	0.838	0.444	0.735 (0.556–0.914)
PCT > 1.0 ng/ml	0.750	0.600	0.871	0.400	
CRP > 10 mg/l	0.919	0.000	0.773	0.000	0.599 (0.421–0.777)
Lactate > 20 mg/dl	0.441	0.900	0.938	0.321	0.672 (0.503–0.841)

In sensitivity analyses using a POC bio-ADM cut-off defined by the batch-specific LOD (> 30 pg/ml or > 45 pg/ml), associations between elevated POC bio-ADM and bacteremia or the diagnosis of any infection remained directionally similar, although effect estimates were attenuated and the association with bacteremia was no longer statistically significant (RR 1.89, 95% CI 0.82–4.36 for bacteremia; RR 1.67, 95% CI 1.21–2.30 for any infection) (supplement S.1). Sensitivity, specificity, PPV and NPV for the batch-specific LOD cut-offs are provided in supplement S.2. The Youden-index-derived POC bio-ADM cut-off (46.1 pg/ml) did not alter patient classification compared with the primary 45 pg/ml cut-off as no POC bio-ADM measurements lay between these values and therefore produced identical diagnostic estimates. At Youden-optimized cut-offs, all four biomarkers showed broadly similar discrimination for bacteremia, with sensitivities around 0.63–0.75 and specificities around 0.58–0.74 (supplement S.2). For any infection, POC bio-ADM at 32 pg/ml achieved a sensitivity of 0.76 and a specificity of 0.80, with a high positive predictive value (0.93) but only moderate negative predictive value (0.47), indicating a rule-in rather than rule-out profile in this high-prevalence cohort. These estimates are exploratory and reflect the limited sample size.

The primary endpoint demonstrated a large effect size with an odds ratio of 5.318 (95% CI: 1.217–23.243) for the association between positive POC bio-ADM and bacteremia. The wide confidence interval reflects the small sample size and suggests a need for larger studies. For attributable mortality, the risk difference of 40.8% (95% CI: 18.1–63.5%) represents a large clinical effect. Given the small number of deaths and baseline differences in age and SOFA scores, these mortality findings should be interpreted with particular caution. To detect the observed odds ratio of 5.3 for bacteremia in futures studies with 80% power and α = 0.05, assuming similar prevalence rates (POC bio-ADM positive: 38%, bacteremia: 34%), approximately 61 patients would be needed. For 90% power, 81 patients would be required.

## Discussion

Early identification and treatment of patients with severe infections, is crucial in the ED. Any delay is linked to unfavourable outcomes [18–22]. Ideally, patients with severe infections should be identified prior to the onset of organ failure, and effective treatment should be initiated promptly. Several studies suggest that bio-ADM may serve as a predictive biomarker, especially in relation to mortality, severity of illness, and the need for organ support in sepsis patients [11, 14, 15, 23–27].

In this single-centre exploratory pilot study, POC bio-ADM was associated with higher rates of bacteremia, the diagnosis of infection, and with increased in-hospital mortality among ED patients with suspected severe infection. In these patients, a positive POC bio-ADM result was associated with an odds ratio for bacteremia of 5.318 (95% CI: 1.217–23.243).

In this small cohort, POC bio-ADM showed diagnostic performance for bacteremia and any infection that was broadly comparable to established biomarkers such as PCT, CRP and lactate when evaluated using AUCs and LOD-based as well as Youden-index optimized cut-offs. At these thresholds, POC bio-ADM tended to have higher specificity and positive predictive value, whereas sensitivity and negative predictive value were in a similar range to PCT and the other biomarkers, and all estimates remained imprecise with wide confidence intervals, underlining their exploratory nature (supplement S.2). Additionally, elevated POC bio-ADM levels were associated with higher in-hospital mortality, while attributable mortality among POC bio-ADM-negative patients was low. Because patients with elevated POC bio-ADM were older and had higher SOFA scores, part of the observed association likely reflects overall severity of illness rather than an effect independent of established clinical scores.

The choice of bacteremia as the primary endpoint was based on its objective and reproducible nature and on its clinical relevance in patients with suspected severe infection in the ED. However, only a subset of patients with severe infection or sepsis are bacteremic, and culture-negative sepsis remains common. Therefore, the diagnosis of any infection was evaluated as secondary endpoint, and elevated POC bio-ADM was associated not only with bacteremia but also with infection in general. These findings support the hypothesis that POC bio-ADM reflects a broader infectious and inflammatory burden rather than the presence of bacteria in the bloodstream alone, although this requires confirmation in larger cohorts.

Bacteremia was diagnosed based on blood cultures, which may include both false positives (contaminants) and false negatives (e.g. prior antibiotic exposure, low-grade bacteremia). Although typical skin flora without compatible clinical or imaging findings were predefined as contaminants and classified as non-bacteremic, residual misclassification is possible. Furthermore, bio-ADM may be elevated in patients with non-infectious conditions involving endothelial dysfunction. Conversely, it may remain low in early or localised infections with limited systemic inflammation. Both sources of misclassification are likely to reduce the strength of the observed association between POC bio-ADM and bacteraemia.

Due to Point-of-Care testing, the results of the bio-ADM test were rapidly available. POC bio-ADM testing may thus influence time-sensitive treatment decisions and aid in the risk stratification of ED patients presenting with symptoms suggestive for severe infections.

These findings support the hypothesis that a two-step diagnostic strategy. Initial screening with NEWS-2 followed by targeted POC bio-ADM testing could be evaluated in future studies as a testable clinical algorithm. In this concept, a broadly sensitive score such as NEWS-2 would serve as a first-line screening tool, and POC bio-ADM would be used as a second-line test to improve specificity and risk stratification. However, this approach requires prospective validation before it can inform antimicrobial or disposition decisions.

With regard to the literature, a meta-analysis of 24 studies including 6730 adult patients found higher levels of midregional pro-adrenomedullin (MR-proADM), another precursor of ADM, in hospitalized non-survivors with sepsis or septic shock. Limitations of the meta-analysis were heterogeneous cohorts in different settings and no comparison of MR-proADM with other biomarkers [16]. Also, in terms of clinical applicability, there are no specific cut-off values of MR-proADM to define patients at risk of adverse outcome. Therefore, the role of MR-proADM as a predictive biomarker for mortality in patients with suspected severe infection remained uncertain, especially in the setting of patients presenting to the ED. In the realm of ED sepsis patients, Lundberg et al. found an association of elevated bio-ADM levels and increased mortality, a higher incidence of multi-organ failure, and ICU admissions [13]. Given the difficulty of identifying sepsis patients in the ED due to imperfection of clinical scores and lack of readily available biomarkers, our study diverges from Lundberg et al.‘s research. The objective of this study was to evaluate the predictive power of POC bio-ADM for bacteremia in patients who present to the ED with a suspected severe infection. The patient population in this study is less preselected than the cohort investigated by Lundberg et al. [13], which more accurately mirrors the reality in the ED, where patients typically present with symptoms rather than a pre-existing diagnosis. The observed mortality rates (26.1% overall, 19.6% attributable) are consistent with European sepsis outcomes reported by Bauer M et al. [28], validating the representativeness of our study population.

In a study involving 312 patients presenting with suspected infection at a German emergency department, Bauer W et al. found that a qSOFA score of ≥ 2 failed to detect 41% of sepsis cases in this cohort, yielding a sensitivity of 57% and a specificity of 83%. In the context of this study, it was noted that 50% of those patients who died during their hospitalization had a qSOFA < 2 at the time of their admission to the ED, indicating a high false negative rate of qSOFA. In contrast, a NEWS-2 ≥ 5 demonstrated a higher sensitivity (96%) but a lower specificity (45%) [29]. Among the biomarkers evaluated in this study, POC bio-ADM exhibited the highest specificity and positive predictive value, which is consistent with the proposed NEWS-2 based two-step triage concept and could provide a rationale for evaluating this algorithm in prospective studies.

Our study excluded patients suspected of having septic shock. This was based on established guidelines that recommend the immediate initiation of antibiotic therapy following microbiological sampling within one hour of presentation [2]. Consequently, waiting for biomarker results is not advised, and biomarkers do not play a role in the acute treatment decision-making process for patients with suspected septic shock.

Additionally, the findings of this study align with other research indicating that an elevated level of bio-ADM is an independent risk factor for increased in-hospital mortality. In contrast to the study by Lundberg et al. [13], which investigated bio-ADM levels in ED sepsis patients defined by Sepsis-2-criteria [30], this study did not find an association between elevated POC bio-ADM levels and increased ICU admission rates. 8.6% of the cohort investigated by Lundberg et al. died within 28 days and non-survivors had higher levels of bio-ADM than survivors. In this study sepsis was defined according to the Sepsis-3-definition [2]. In contrast to the study of Lundberg et al., 76.6% of the examined cohort in this study met the sepsis criteria, but a higher in-hospital mortality rate (26.1%) was observed. Considering an average 30-day mortality rate of 32.5% among septic patients in Europe, the outcome data from this study align with the findings of the systematic review by Bauer M et al. [28].

A limitation of this study is the size of the study cohort. The small sample size (*n* = 47) results in wide confidence intervals around the effect estimates including the primary endpoint odds ratio. These wide intervals limit the precision of our findings. Further limitations of this study include its single-centre design, recruitment limited to certain working hours, and exclusion of patients with septic shock introducing selection, spectrum, and verification biases and preclude precise estimation of diagnostic accuracy. Bacteremia and the diagnosis of any infection were ascertained from routine documentation and discharge summaries, so some misclassification of infection status cannot be excluded. Despite blinded adjudication of attributable in-hospital mortality by two physicians and resolution of the single discrepant case by a third reviewer, some misclassification of cause of death may also persist. In addition, enrolment had to be stopped when the manufacturer discontinued production of the POC bio-ADM test kits and no further kits were available, which restricted the achievable sample size and contributed to the imprecision of effect estimates. Accordingly, all estimates of diagnostic performance, including AUCs and operating characteristics at LOD-based and Youden-optimized cut-offs, should be interpreted as exploratory and are insufficient to establish a definitive ranking between biomarkers.

Bacteremia was diagnosed based on blood culture results, which may include false positives or false negatives. The change in POC bio-ADM assay LOD and retrospective harmonisation to a 45 pg/ml cut-off may have led to misclassification. Future work should standardise assay performance and prospectively define thresholds, and our LOD-based sensitivity analyses can only provide preliminary, hypothesis-generating information in this regard. Multivariable models are underpowered and at risk of overfitting. These analyses are presented solely as exploratory and hypothesis-generating.

These limitations should be taken into account when interpreting the results of this study. Future research should aim to address these limitations to provide more robust and generalizable findings.

## Conclusion

In this single centre exploratory pilot study, elevated POC bio-ADM was associated with bacteremia, the diagnosis of infection, and higher in hospital mortality among ED patients with suspected severe infection. These results suggest that POC bio-ADM merits further evaluation as a component of ED risk stratification algorithms, but the small, selected sample and wide confidence intervals mean that no clinical implementation can be recommended at this stage. Larger, multicentre diagnostic studies with standardised assay thresholds are required.

## Supplementary Information

Below is the link to the electronic supplementary material.

Supplementary Material 1

## Data Availability

The data that support the findings of this study are not openly available due to reasons of sensitivity and are available from the corresponding author upon reasonable request. Data are located in controlled access data storage at University Hospital Regensburg.
