# Supplementary material for: Bioactive adrenomedullin as a point-of-care biomarker in emergency department patients with suspected severe infections: an exploratory analysis
Source: BMC Infect Dis. 2026 Feb 28;26:715. doi: 10.1186/s12879-026-12951-1 (PMC13049759; doi:10.1186/s12879-026-12951-1)
Supplement: Supplementary file 1 — Supplementary Material 1 [file 12879_2026_12951_MOESM1_ESM.docx]

**Supplement**

|  | Biomarker negative | Biomarker positive | RR | p-value |
| --- | --- | --- | --- | --- |
| **Blood cultures positive** |  |  |  |  |
| Bio-ADM [n = 47] (cut-off > batch LOD) | **24.0%** (11.5-43.4%) | **45.5%** (26.9-65.3%) | **1.89** (0.82-4.36) | **0.121** |
| **Any Infection** |  |  |  |  |
| Bio-ADM [n = 47] (cut-off > batch LOD) | **60.0%** (40.7-76.6%) | **100%** (85.1-100%) | **1.67** (1.21-2.30) | **< 0.001** |

S.1: Rates of positive blood cultures and infections of any type in relation to positive or negative test results for bio-ADM with batch specific LOD cut-off; RR (relative risk); 95% CI in round brackets

|  | **Sensitivity** | **Specificity** | **PPV** | **NPV** | **p-value** |
| --- | --- | --- | --- | --- | --- |
| **Bacteremia** |  |  |  |  |  |
| Bio-ADM > batch LOD | 0.625 | 0.613 | 0.760 | 0.455 | 0.121 |
| Bio-ADM > 46.1 pg/ml | 0.625 | 0.742 | 0.556 | 0.793 | 0.014 |
| PCT > 2.07 ng/ml | 0.750 | 0.667 | 0.545 | 0.833 | 0.007 |
| CRP > 203 mg/l | 0.688 | 0.581 | 0.458 | 0.783 | 0.081 |
| Lactate > 18 mg/dl | 0.733 | 0.621 | 0.500 | 0.818 | 0.026 |
| **Any Infection** |  |  |  |  |  |
| Bio-ADM > batch LOD | 0.595 | 1.000 | 1.000 | 0.400 | < 0.001 |
| Bio-ADM > 32 pg/ml | 0.757 | 0.800 | 0.933 | 0.471 | < 0.001 |
| PCT > 0.73 ng/ml | 0.833 | 0.600 | 0.882 | 0.500 | 0.006 |
| CRP > 220 mg/l | 0.514 | 0.800 | 0.905 | 0.308 | 0.077 |
| Lactate > 31 mg/dl | 0.353 | 1.000 | 1.000 | 0.313 | 0.028 |

S.2: Biomarker performance at bio-ADM batch LOD and Youden-optimized cut-offs

S.3: Receiver operating characteristic (ROC) curves of bio-ADM, PCT, CRP and lactate for bacteremia

| 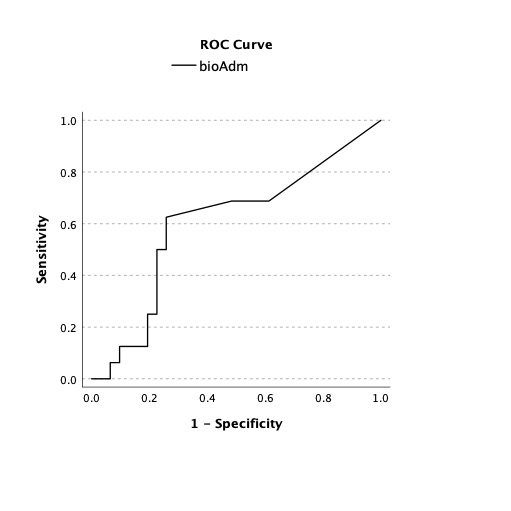 | 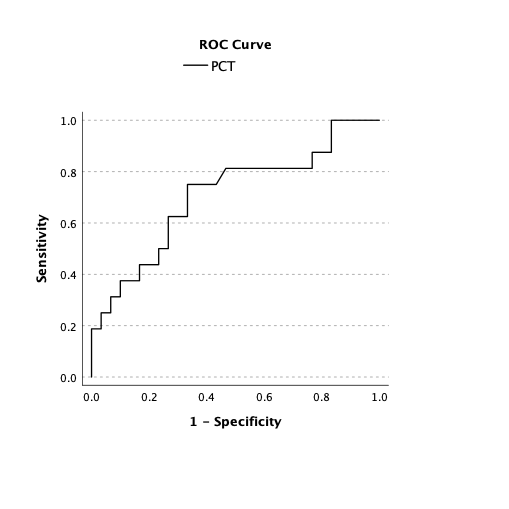 |
| --- | --- |
| 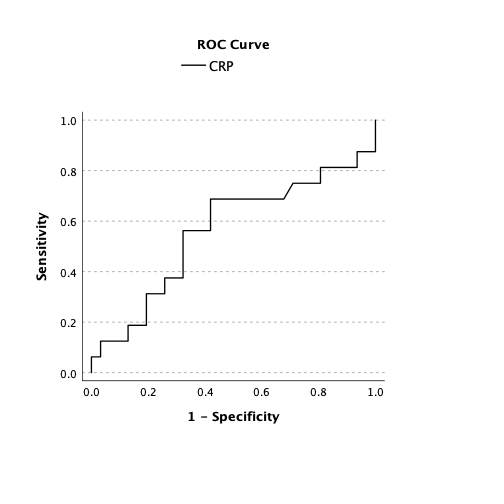 | 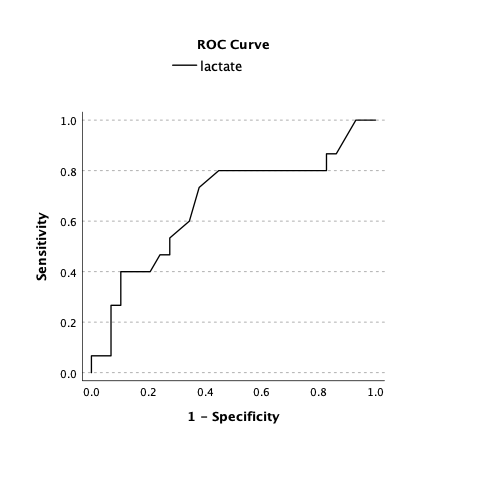 |

S.4: Receiver operating characteristic (ROC) curves of bio-ADM, PCT, CRP and lactate for any infection.

| 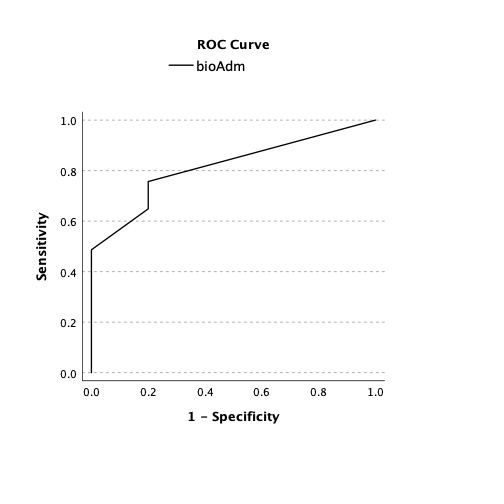 | 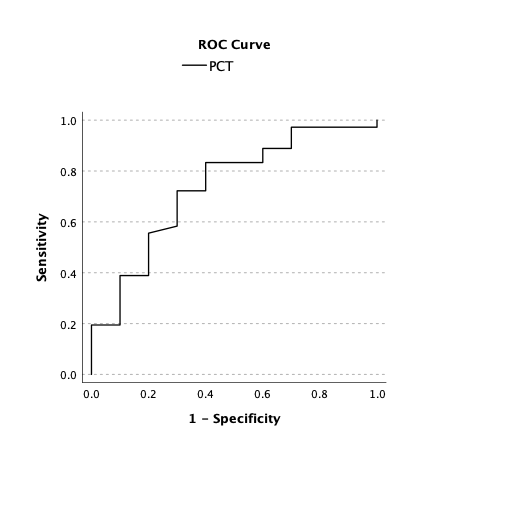 |
| --- | --- |
| 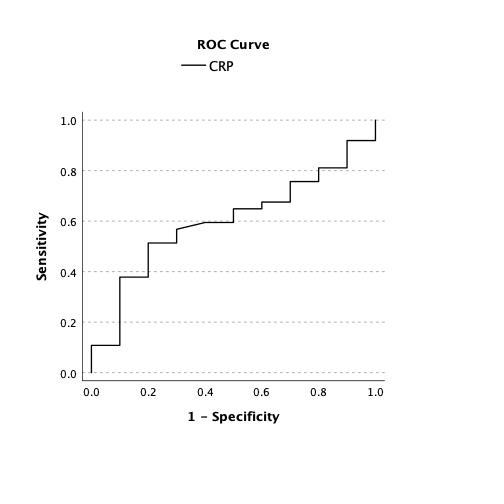 | 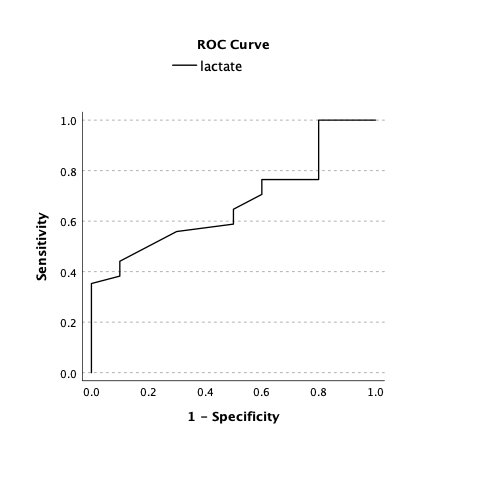 |

S. 5: The STARD 2015 list*

| Section and topic  p. (page) | No | Item |
| --- | --- | --- |
| **Title or abstract** | | |
| p. 1 | 1 | Identification as a study of diagnostic accuracy using at least one measure of accuracy (such as sensitivity, specificity, predictive values, or AUC) |
| **Abstract** | | |
| p. 1-2 | 2 | Structured summary of study design, methods, results, and conclusions (for specific guidance, see STARD for Abstracts) |
| **Introduction** | | |
| p. 2-4 | 3 | Scientific and clinical background, including the intended use and clinical role of the index test |
| p. 4 | 4 | Study objectives and hypotheses |
| **Methods** | | |
| p. 4-5 | 5 | Whether data collection was planned before the index test and reference standard were performed (prospective study) or after (retrospective study) |
| p. 5 | 6 | Eligibility criteria |
| p. 5 | 7 | On what basis potentially eligible participants were identified (such as symptoms, results from previous tests, inclusion in registry) |
| p. 5 | 8 | Where and when potentially eligible participants were identified (setting, location, and dates) |
| p. 4 | 9 | Whether participants formed a consecutive, random, or convenience series |
| p. 5-6 | 10a | Index test, in sufficient detail to allow replication |
| p. 6 | 10b | Reference standard, in sufficient detail to allow replication |
| p. 6 | 11 | Rationale for choosing the reference standard (if alternatives exist) |
| p. 5-6 | 12a | Definition of and rationale for test positivity cut-offs or result categories of the index test, distinguishing pre-specified from exploratory |
| p. 7 | 12b | Definition of and rationale for test positivity cut-offs or result categories of the reference standard, distinguishing pre-specified from exploratory |
| p. 6-7 | 13a | Whether clinical information and reference standard results were available to the performers or readers of the index test |
| p. 6-7 | 13b | Whether clinical information and index test results were available to the assessors of the reference standard |
| p. 8 | 14 | Methods for estimating or comparing measures of diagnostic accuracy |
| p. 5-6 | 15 | How indeterminate index test or reference standard results were handled |
| p. 5 | 16 | How missing data on the index test and reference standard were handled |
| p. 7 | 17 | Any analyses of variability in diagnostic accuracy, distinguishing pre-specified from exploratory |
| p. 4-5 | 18 | Intended sample size and how it was determined |
| **Results** | | |
| p. 9, Fig. 1 | 19 | Flow of participants, using a diagram |
| p. 10-11, Tab. 1 | 20 | Baseline demographic and clinical characteristics of participants |
| p. 10-11, Tab. 1 | 21a | Distribution of severity of disease in those with the target condition |
| p. 10 | 21b | Distribution of alternative diagnoses in those without the target condition |
| p. 6 | 22 | Time interval and any clinical interventions between index test and reference standard |
| p. 12, Tab. 2 | 23 | Cross tabulation of the index test results (or their distribution) by the results of the reference standard |
| p. 15-16, Tab. 7 | 24 | Estimates of diagnostic accuracy and their precision (such as 95% confidence intervals) |
| not applicable | 25 | Any adverse events from performing the index test or the reference standard |
| **Discussion** | | |
| p. 21-22 | 26 | Study limitations, including sources of potential bias, statistical uncertainty, and generalisability |
| p. 19-20 | 27 | Implications for practice, including the intended use and clinical role of the index test |
| **Other information** | | |
| p. 5, p. 22, pilot-study | 28 | Registration number and name of registry |
| p. 23 | 29 | Where the full study protocol can be accessed |
| p. 23 | 30 | Sources of funding and other support; role of funders |

Bossuyt PM, Reitsma JB, Bruns DE, et al (2015) STARD 2015: an updated list of essential items for reporting diagnostic accuracy studies. BMJ 351:h5527. https://doi.org/10.1136/bmj.h5527
